# Nursing professional practice environments and job satisfaction: process and outcome interfaces

**DOI:** 10.1590/0034-7167-2023-0544

**Published:** 2025-06-27

**Authors:** Alessandro Rodrigues Perondi, Letícia de Lima Trindade, Olga Maria Pimenta Lopes Ribeiro, Jane Tavares Gomes, Jouhanna do Carmo Menegaz, Simone Coelho Amestoy, José Luis Guedes dos Santos, João Miguel Almeida Ventura-Silva

**Affiliations:** I Universidade Comunitária da Região de Chapecó. Chapecó, Santa Catarina, Brazil; II Escola Superior de Enfermagem do Porto. Porto, Portugal; III Universidade do Estado de Santa Catarina. Chapecó, Santa Catarina, Brazil; IV Universidade Federal do Vale do São Francisco. Petrolina, Pernambuco, Brazil; V Universidade Federal de Santa Catarina. Florianópolis, Santa Catarina, Brazil; VI Escola Superior de Saúde Norte da Cruz Vermelha Portuguesa. Oliveira de Azeméis, Portugal

**Keywords:** Nursing, Working Environment, Professional Practice, Job Satisfaction, Hospitals, Enfermería, Ambiente de Trabajo, Practica Profesional, Satisfacción en el Trabajo, Hospitales

## Abstract

**Objectives:**

to assess the process and outcome of professional nursing practice environments and their relationship with job satisfaction.

**Methods:**

a cross-sectional study conducted in eight hospitals in southern Brazil. Participants were 291 nurses who answered a sociodemographic/professional questionnaire, the Scale for the Environment Evaluation of Professional Nursing Practice, and the Job Satisfaction Survey. Descriptive and inferential statistical analysis was used.

**Results:**

in process, theoretical and legal support of professional practice and care planning, evaluation and continuity presented the highest scores, while interdependent practices in professional practice obtained the lowest. Outcome was favorable to quality of care, with systematic assessment of nursing care and indicators being very favorable, but systematic assessment of nurses’ performance and supervision was moderate. Process had a large effect, and outcome had a medium effect on the total job satisfaction score.

**Conclusions:**

the process and outcome elements indicate how to promote professional satisfaction and nursing quality of care.

## RESUMEN

**Objetivos:**

evaluar el proceso y resultado de los ambientes de práctica profesional de enfermería y su relación con la satisfacción laboral.

**Métodos:**

estudio transversal, realizado en ocho hospitales del sur de Brasil. Participaron 291 enfermeros que completaron un cuestionario sociodemográfico/profesional, *Scale for the Environment Evaluation of Professional Nursing Practice* y Escala de Satisfacción Laboral. Se utilizó análisis estadístico descriptivo e inferencial.

**Resultados:**

en el proceso, subsidios teórico-legales y planificación, evaluación y continuidad de la atención presentaron los puntajes más altos, mientras que las prácticas interdependientes obtuvieron los peores. El resultado fue favorable a la calidad de la atención, siendo muy favorable la evaluación sistemática de la atención y los indicadores, pero moderada la evaluación sistemática del desempeño y la supervisión. Hubo un gran efecto del proceso y el promedio del resultado en el puntaje total de satisfacción laboral.

**Conclusiones:**

los elementos del proceso y resultado que indican cómo promover la satisfacción profesional y la calidad de la atención de enfermería.

## INTRODUCTION

Quality management of healthcare services has been a topic of study over the years, and is considered a priority in the search for improving patient safety and organizational sustainability. This issue becomes a challenge for nursing leaders, due to the complexity involved in professional practice environments and care processes^(1)^.

The expression “professional nursing practice environment” is used internationally to define a set of concrete and abstract organizational characteristics that facilitate or restrict professional nursing practice in delivering high-quality care^(2)^.

It is recognized that nurses and work environments play a fundamental role in ensuring the safety and quality of care provided, recognizing that some attributes, such as professional autonomy, control over nurses’ professional practice, cordial professional relationships between doctors and nurses and organizational support, are essential elements for building a favorable or unfavorable environment^(3)^.

The literature shows that favorable nursing professional practice environments have been associated with greater professional satisfaction^(4)^, low levels of burnout syndrome and emotional exhaustion^(1)^, reduced intention to leave the job^(5)^, improved safety climate and quality of healthcare^(6,7)^, reduced lack of nursing care^(8)^, lower absenteeism rates and reduced iatrogenesis^(3,6)^.

As part of efforts to improve patient safety and quality of care, professional organizations such as the Institute of American and the Nurses Credentialing Center (ANCC) have emphasized the promotion of nursing work environments^(3)^. The World Health Organization, in its report on the state of nursing in the world, recalls the need for countries to provide environments that are favorable to nursing practice, in order to attract, retain, and motivate this workforce^(9)^, since these professionals are decisive in the quality and safety of care provided to clients, families, and/or caregivers. In May 2022, the International Labor Organization (ILO), in Article 7, included safe and healthy working conditions in its framework of fundamental principles and rights at work^(10)^.

According to Donabedian’s theoretical model^(11)^, some factors contribute to improving health quality and, consequently, to qualifying practice environments. For its assessment, a triad consisting of structure, process and outcome is established. Structure is understood as physical, human, material, financial and equipment resources necessary for healthcare; process refers to activities involving healthcare professionals and users, including diagnosis, treatment, and ethical aspects of professional relationships; and outcome corresponds to the final product of the care provided, considering health, satisfaction of standards and user expectations^(11)^. The process refers to factors related to the performance of activities inherent to nursing care design and provision. Outcome assesses desirable or undesirable changes in relation to care, clients and nurses^(12)^.

Despite the advances in knowledge production on this topic, the literature has shown that most studies use assessment scales derived from the Nursing Work Index, bringing with them the limitation of being essentially focused on the organization’s structural conditions and superficially on the process and outcome components^(12)^.

In this regard, nursing practice environment studies that contemplate the process and outcome dimensions emerge as an alternative to contribute to the understanding of the aspects that interfere with the exercise of professional practice and that, consequently, can have repercussions on patient care^(1)^, with professional satisfaction also being an object of interest. This corresponds to professionals’ perception about the work they perform and the environment in which it is produced^(13)^.

Job satisfaction can influence the quality of care provided, reduce the level of burnout, decrease the mortality rate and, consequently, decrease turnover and absenteeism, promote interest in work and the quality of interpersonal relationships^(14)^, with the impact of healthy work environments on nurses’ satisfaction and retention being the focus of studies in the last decade^(3,15,16)^.

In Brazil, it is considered pertinent to develop investigations that identify several factors, not only organizational ones, which may have an impact on care provision, with a view to supporting decision-making by leaders, in the search for continuous improvement of processes and better conditions for nursing team work^(2)^.

## OBJECTIVES

To assess the process and outcome of professional nursing practice environments and their relationship with job satisfaction.

## METHODS

### Ethical aspects

The study was approved by the Research Ethics Committee and followed all the guidelines of Resolution 466/2012. Participants signed the Informed Consent Form.

### Study design, period and location

This is a cross-sectional and quantitative study, guided by the STrengthening the Reporting of OBservational studies in Epidemiology (STROBE) tool. It was carried out in eight hospitals in southern Brazil, intentionally chosen because they are reference hospitals in the provision of public beds and in medium and high complexity care.

### Population and sample definition

Non-probabilistic convenience sampling was used, considering a 50% heterogeneity, a 95% Confidence Interval, and a 5% sampling error, which resulted in a minimum sample of 232 participants, proportionally stratified by each hospital. However, data collection totaled a sample of 291 nurses (Chart 1), considering the inclusion criteria: a) working as a nurse in the study hospitals; b) having a period of experience in the unit equal to or greater than three months; c) being working during the collection period, i.e., not being on leave for any reason. Nurses from all sectors of selected institutions, who provided direct care to patients, in different work shifts, participated in the study.

**Chart 1:** Data identifying the scenario and number of study participants (N=232), Southern region, Brazil, 2021

Identification	Population/nurses	Sample
Hospital A	68	27
Hospital B	28	11
Hospital C	39	16
Hospital D	40	16
Hospital E	179	71
Hospital F	77	30
Hospital G	129	51
Hospital H	25	10
**Total**	**581**	**232**

*Source: http://cnes.datasus.gov.br, March, 2021.*

### Instruments used to collect information

Data collection took place from July to October 2021, through on-site visits to selected institutions. A structured questionnaire consisting of three parts was used. In the first part, information was sought on sociodemographic and professional characteristics (age, sex, marital status, professional training, type of institution - public or private, work unit, professional condition - care or coordination, length of professional practice, length of work in the institution and in the unit).

The second part contained the Scale for the Environment Evaluation of Professional Nursing Practice (SEE-Nursing Practice)^(12)^, linguistically adapted to Brazilian Portuguese for this study. The SEE-Nursing Practice consists of three subscales: SEE-Nursing Practice - Structure; SEE-Nursing Practice - Process; and SEE-Nursing Practice - Outcome.

In this study, the SEE-Nursing Practice - Process and the SEE-Nursing Practice - Outcome were used. The SEE-Nursing Practice - Process consists of 37 items, divided into six dimensions: 1 - collaboration and teamwork (9 items); 2 - strategies for ensuring quality in professional practice (7 items); 3 - autonomous practices in professional practice (7 items); 4 - care planning, evaluation and continuity (6 items); 5 - theoretical and legal support of professional practice (4 items); and 6 - interdependent practices in professional practice (4 items). The SEE-Nursing Practice - Outcome includes 13 items, divided into two dimensions: 1 - systematic assessment of nursing care and indicators (7 items); 2 - systematic assessment of nurses’ performance and supervision (6 items)^(12)^.

The internal consistency of the Brazilian version obtained a Cronbach’s alpha coefficient of 0.95 for SEE-Nursing Practice - Process and 0.94 for SEE-Nursing Practice - Outcome. The response is measured for each item on a Likert-type scale with five options, where one corresponds to “never”, two to “rarely”, three to “sometimes”, four to “often”, and five to “always”^(12)^.

The third part of the study used the Job Satisfaction Survey (JSS), a reduced version^(15)^, which aims to identify the degree of satisfaction of employees in relation to their work environment. The JSS has 15 items and is subdivided into five dimensions, each consisting of three items: satisfaction with coworkers (α = 0.86); satisfaction with pay (α = 0.92); satisfaction with management (α = 0.90); satisfaction with the nature of work (α = 0.82) and satisfaction with promotions (α = 0.87). Participants’ responses are obtained using a seven-point scale (1 = completely dissatisfied; 2 = very dissatisfied; 3 = dissatisfied; 4 = indifferent; 5 = satisfied; 6 = very satisfied; 7 = completely satisfied), and the analysis of scale results is based on the mean score of each of the five dimensions^(17)^.

### Data treatment and analysis

The collected data were analyzed using the Statistical Package for the Social Sciences (SPSS) version 21.0. Categorical variables were described by absolute and relative frequencies. Regarding the SEE-Nursing Practice and its subscales, the higher the score, the more favorable the process and outcome are to quality of care. To analyze the results, the mean and standard deviation of items were described, and the following criteria were used^(12)^: score < 35% - component of professional nursing practice environment not very favorable to quality of care; between 35% and 55% - component of professional nursing practice environment moderately favorable to quality of care; between 55% and 75% - component of professional nursing practice environment favorable to quality of care; and > 75% - component of professional nursing practice environment very favorable to quality of care.

To express the JSS outcome, it was considered that the higher the mean score, the greater the degree of employee contentment or satisfaction with that dimension. Values between 5 and 7 indicate satisfaction; values between 1 and 3.9 indicate dissatisfaction; and values between 4 and 4.9 indicate indifference^(17)^.

To compare means, Student’s t-test for independent samples or Analysis of Variance (ANOVA) were applied. Associations between continuous variables were assessed by Pearson’s or Spearman’s correlation tests, considering correlation coefficient values below 0.3 as small effect, between 0.30 and 0.49, as medium effect, and between 0.50 and 1, as large effect. To control for confounding factors, Multivariate Linear Regression analysis was used. The regression coefficient or slope (b), which measures the effect on the outcome for each increase of one unit of the factor, and the 95% Confidence Interval were calculated. The criterion for entering the variable into the model was that it presented a p-value<0.20 in bivariate analysis. The significance level adopted was 5% (p≤0.05).

## RESULTS

A total of 291 nurses participated in the study, of whom 251 (86.3%) were female and 216 lived with a partner (74.2%). The level of education showed that 176 (60.5%) had a specialization, 100 (34.4%) were only graduates and 15 (5.1%) had a master’s or doctoral degree. The mean age was 34.2 ± 8.4 years; the median length of service in the profession was six years (2-12); and the median length of service in the current service was two (1-7).

There was a higher prevalence of nurses who worked in public institutions (n=114/39.2%), preceded by philanthropic institutions (n=101/4.7%) and private institutions (n=76/26.1%). Of these, 212 (72.9%) worked in care and 79 (27.1%) were responsible for, in addition to care, coordination. Table 1 presents the results of the assessment of the process dimension in the environments studied.

**Table 1:** Assessment of the process dimension of professional nursing practice environments, Southern region, Brazil, 2021

Process	Mean ± SD	SF^*^	MDF^*^	F^*^	VF^*^
		n (%)	n (%)	n (%)	n (%)
Collaboration and teamwork	68.6 ± 16.8	8 (2.7)	48 (16.5)	145 (49.8)	90 (30.9)
Strategies for ensuring quality in professional practice	65.1 ± 18.1	15 (5.2)	71 (24.4)	130 (44.7)	75 (25.8)
Autonomous practices in professional practice	68.2 ± 15.2	4 (1.4)	53 (18.2)	160 (55.0)	74 (25.4)
Care planning, evaluation and continuity	71.4 ± 16.1	6 (2.1)	44 (15.1)	135 (46.4)	106 (36.4)
Theoretical and legal support of professional practice	78.9 ± 15.1	2 (0.7)	18 (6.2)	133 (45.7)	138 (47.4)
Interdependent practices in professional practice	31.1 ± 17.5	171 (58.8)	94 (32.3)	24 (8.2)	2 (0.7)
Total	65.4 ± 11.6	2 (0.7)	57 (19.6)	170 (58.4)	62 (21.3)

* *SF - slightly favorable; MDF - moderately favorable; F - favorable; VF - very favorable.*

The table reveals that theoretical and legal support of professional practice (mean=78.9 ± 15.1) and care planning, evaluation and continuity (mean=71.4 ± 16.1) were the items that statistically presented the highest scores. The worst score was observed in interdependent practices in professional practice (31.1 ± 17.5). The general assessment of the process was favorable to quality of care (mean=65.4 ± 11.6).

Table 2 presents the mean scores of the outcome dimension present in the professional nursing practice environments researched.

**Table 2:** Assessment of the outcome dimension of professional nursing practice environments, Southern region, Brazil, 2021

Outcome	Mean ± SD	SF^*^	MDF^*^	F^*^	VF^*^
		n (%)	n (%)	n (%)	n (%)
Systematic assessment of nursing care and indicators	64.2 ± 19.8	22(7.6)	75 (25.8)	125(43.0)	69 (23.7)
Systematic assessment of nurses’ performance and supervision	53.2 ± 21.7	65 (22.3)	99 (34.0)	88 (30.2)	39 (13.4)
Total	59.1 ± 19.3	35 (12.0)	86 (29.6)	116(39.9)	54 (18.6)

* *SF - slightly favorable; MDF - moderately favorable; F - favorable; VF - very favorable.*

Systematic assessment of nursing care and indicators presented a higher score (mean=64.2 ± 19.8). Systematic assessment of nurses’ performance and supervision (mean=53.2 ± 21.7) was moderately favorable, however the general assessment of the outcome dimension was also favorable to quality of care (mean=59.1 ± 19.3).

An association was identified between sample characterization variables and the scores of the process and outcome dimensions in sex (p=0.267 and p=0.920), marital status (p=0.991 and p=0.720), academic degree (p=0.190 and p=0.024), type of institution (p=0.979 and p=0.824) and professional status (p=0.306 and p=0.761).

Inverse and statistically significant associations between process and outcome were observed with age (-0.171; p=0.003 and -0.174; p=0.003), length of service in the profession (-0.217; p<0.001 and -0.217; p<0.001) and also length of service in the service (-0.185; p<0.002 and -0.153; p<0.009), respectively. It was demonstrated that the greater the age and the length of service in the profession and service, the lower the score for the process and outcome dimensions. However, a significant association was also revealed between academic level and the outcome dimension, and as the level of education increases, the score for this dimension decreases.

To control for confounding factors, variables with p <0.20 were entered into a Multivariate Linear Regression model. After adjustment by the multivariate model, the length of service in the profession remained an independent factor associated with process and outcome, and the longer the length of service in the profession, the lower the scores in these dimensions (Table 3).

**Table 3:** Characterization of variables according to the Multivariate Linear Regression model, Southern region, Brazil, 2021

Variables	Process		Outcome	
	b (95% CI)	*p* value	b (95% CI)	*p* value
Age (years)^*^	0.03 (-0.20 a 0.25)	0.822	0.02 (-0.35 a 0.40)	0.900
Academic degree				
Graduation	0.00	-	0.00	-
Specialization	-0.13 (-3.13 a 2.87)	0.932	-1.94 (-6.90 a 3.02)	0.442
Masters/Doctoral degree	-0.80 (-7.29 a 5.70)	0.809	-6.59 (-17.3 a 4.17)	0.229
Length of time working in the profession (years)^**^	-0.38 (-0.72 a -0.05)	0.025	-0.65 (-1.21 a -0.10)	0.022
Length of service (years)^**^	-0.06 (-0.41 a 0.29)	0.720	0.06 (-0.52 a 0.64)	0.836

* Pearson’s correlation coefficient;

** Spearman’s correlation coefficient.

Concerning the JSS, the overall score demonstrated indifference (mean 4.60 ± 0.78). The scores of the isolated dimensions demonstrated satisfaction with management (mean 5.34 ± 1.06), with coworkers (mean 4.94 ± 0.87) and with the nature of work (mean 4.91 ± 0.84), indifference with promotions (mean 4.20 ± 1.19) and dissatisfaction with pay (mean 3.58 ± 1.32).

Table 4 presents the association between items with p≤0.001 of EST with the SEE-Nursing Practice process and outcome dimensions. A large effect was observed in the process dimension and a medium effect in the outcome dimension, with the total JSS score.

**Table 4:** Correlation between the Job Satisfaction Survey and the SEE-Nursing Practice process and outcome dimensions through Pearson’s correlation coefficient, Southern region, Brazil, 2021

Job Satisfaction Survey dimensions	SEE-Nursing Practice	
	Process	Outcome
Satisfaction with coworkers	0.304^b^	0.192^a^
Satisfaction with pay	0.310^b^	0.404^b^
Satisfaction with management	0.341^b^	0.270^a^
Satisfaction with the nature of work	0.364^b^	0.284^a^
Satisfaction with promotions	0.449^b^	0.435^b^
Total	0.553^c^	0.490^b^

a small effect;

b medium effect;

c large effect.

## DISCUSSION

Researchers’ interest in exploring and improving the nursing practice environment is not new. The American Association of Critical Care Nurses published and revised standards for establishing and maintaining healthy work environments, updated in 2016^(16)^. Since then, the literature^(18,19)^ has demonstrated that a favorable nursing practice environment is a determining factor for quality of care as well as for obtaining better results for clients, nurses, and institutions, which culminated in its prioritization last year by the ILO^(9)^.

The demographic and labor findings revealed a profile similar to other studies in national and international contexts^(12)^ and to a study carried out in the country by the Federal Nursing Council^(20)^.

The study showed that 60.5% of nurses had specialization, and this finding was significantly associated with the outcome dimension, and that as the level of education increases, the score for this dimension decreases. This dimension assesses desirable or undesirable changes in relation to care, clients, and nurses^(11)^. An increase in the level of education has been associated with a decrease in the mortality rate in hospitals in Belgium^(21)^.

High levels of education are positively related to productivity, since education contributes to increase knowledge, skills, attitudes and behaviors essential for carrying out nursing practice activities^(22)^. Higher education is related to the ability to better assess a situation before acting^(23)^, and investment in training is clearly an important aspect to promote the quality of results in nursing care, which also has the potential to increase criticality and demands on workers. A study also discusses the difficulty for training to ensure a practice that is continually based on evidence; this involves the ability to research and openness to using evidence in practice, which also contributes to changes in environments^(24)^.

Professionals had a median of six years of experience in the profession and two years of experience in their current service, characteristics similar to other studies^(2,14)^, further supporting the data printed by the nursing profile in Brazil^(20)^.

The length of time working in the profession showed an inverse and statistically significant association with the process and outcome dimensions, and the longer the time working in the profession, the lower the scores in these dimensions. The wear and tear over the years and the complexity of professional practice during the pandemic may have contributed to participants having a worse perception of the activities performed and a worse final product of the care provided. In contrast, work experience is a process of developing expertise, since the more work experiences, the more opportunities one has to acquire technical and other skills to perform one’s tasks effectively. Work experience also contributes to a positive work environment, including increased patient safety^(25)^. In this direction, continuing education and permanent education emerge in services that need to consider that, in care contexts, different generations of workers work and the aging of the workforce itself. An environment that empowers and motivates nurses is necessary to sustain the nursing workforce, promoting autonomy in decision-making and concern with workforce exhaustion^(15)^. Knowing how to combine nurses’ knowledge, experiences and innovations brought by newcomers to the profession constitutes a challenge for the profession in the current context.

Public and/or philanthropic institutions comprised 73.9% of professionals involved in the study. According to researchers^(25)^ in Brazil, the public sector has emerged as the largest and most important employer in the category, since 58.9% of the nursing workforce is linked to this sector. However, it is worth noting that the characteristics of the environment in public hospitals and/or those financed by the Brazilian Health System were precarious when compared to those in private hospitals^(26)^.

Underfunding of public hospitals may contribute to inadequate provision of features such as support services, continuing education programs, staffing and career development in these institutions^(25)^.

Participants worked predominantly in care (72.9%), similar to the findings of another study in three university hospitals in southern and southeastern Brazil^(27)^ and of research in a university hospital in São Paulo^(24)^. Nursing is an essential science and profession, considered central to the structure of healthcare professions in Brazil and worldwide. This sociological notion of essentiality makes it possible to work in the various dimensions of health, with a very strong appreciation of care^(25)^.

The devaluation of the role of nursing has been constantly highlighted by international organizations. Nursing professionals are often poorly paid and suffer from gender inequality and precarious working conditions^(28)^, with implications for job satisfaction and the tendency for profession turnover and abandonment^(15)^.

The overall assessment of the process dimension was favorable to quality of care. This dimension assesses factors related to the performance of activities inherent to nursing care design and provision^(12)^. The item related to interdependent practices in professional practice had the worst score. Interdependence is observed in the need for a group of workers to act together to achieve a common goal. An environment unfavorable to interdependent practices is considered to indicate the strong power relations still prevalent in hospitals and the existence of care models that do little to favor interprofessionality.

Conflicting relationships between professionals, especially due to the centrality of valuing medical knowledge, have been described in several studies^(2,13)^, with repercussions on job dissatisfaction and, consequently, negative effects on the quality of work environments.

On the other hand, the overall process score demonstrated a significant association with the total JSS score, indicating that environments that present better assessments on the process dimension present better overall job satisfaction. Professional autonomy and harmonious relationships are practices with the greatest positive predictors in favoring nurses’ professional practice and improving satisfaction in their work environment^(15,29)^.

In the analysis of the outcome dimension, the overall assessment was also favorable to quality of care. This dimension assesses desirable or undesirable changes in relation to care, clients, and nurses^(12)^. This factor is composed of two items, namely systematic assessment of nursing care and indicators and systematic assessment of nurses’ performance and supervision.

Systematic assessment should be understood as a management tool for nurses, which has the potential to generate transformations through gradual and continuous processes of change, aiming at comprehensive care and expanding professional participation^(30)^. However, difficulties in fulfilling exclusive duties, such as care management and the nursing process, are related to staff sizing, with implications for absenteeism, and are fundamental aspects for the quality of the professional nursing environment^(28,31)^.

Thus, the importance of sizing nursing staff is also reinforced, with important aspects regarding the workforce’s quantitative and qualitative aspects, since quality and continuity of care depend on the availability of the workforce and also on their technical capacity for work, i.e., on their professional training, which involves investment in continuing and permanent education in institutions.

The characteristics and attributes identified in nursing practice, including the emphasis on continuous learning and interpersonal relationships, have a profound impact on nursing. Nurses need to demonstrate adaptability, compassion, and patient-centeredness in an ever-evolving healthcare environment^(32)^.

Hence, it is also worth mentioning the growing demand for mastery of rapidly expanding health technologies, which increase demands on nurses’ work, require removing nurses from direct patient care areas and cause some experienced nurses to struggle with technology and abandon the profession due to the difficulty in mastering the technologies^(15)^. These can generate professional satisfaction and dissatisfaction as well as change the professional-patient interaction.

Overall satisfaction showed indifference, however the analysis of isolated dimensions showed dissatisfaction with pay. Researchers have discussed remuneration as the most important domain for nursing team professional satisfaction^(13)^. The establishment of a minimum wage for nursing is currently underway through Bill 2.564 of 2020 and Constitutional Amendment Bill 11 of 2022, both recently approved by the Senate and the Federal Chamber, but with obstacles to its implementation. The absence of a minimum wage and a weekly working day defined by law leads to the team’s work process being organized by accelerated rhythms, long working hours, accumulation of functions, varied employment relationships, fragmentation of care practices, social devaluation, low wages and submission to the repressive and authoritarian conduct of a rigid and vertical hierarchy^(33)^.

The findings also indicated satisfaction with management, coworkers and the nature of work. A good relationship with coworkers and management is known to be a determining factor in job satisfaction. Satisfaction with management is related to the degree to which managers’ professional ability is recognized by subordinates. Satisfaction with coworkers reflects the extent to which workers agree with the collaboration, friendship, trust and relationship established with others^(17)^.

It is worth highlighting that the activities/tasks of hospital nurses are characterized by administrative and managerial assistance, especially regarding the execution of procedures, as they provide material and human resources, plan and supervise the assistance performed by nursing technicians, describe routines, organize the work process and promote in-service education^(34,35)^. However, organizational priorities undergo changes during the pandemic period^(36)^. Satisfaction with this item may signal that the study environments offer the necessary conditions for performing activities with quality and safety.

### Study limitations

The study has limitations, such as data collection, developed in a pandemic context, which may have influenced participants’ response. Longitudinal investigations that systematically assess nursing professional practice environments, in addition to nurses’ professional satisfaction, are relevant.

### Contributions to nursing

The findings indicate the dimensions that compromise the quality of nursing practice environments in hospitals. They reinforce the need for actions that favor nurses’ professional practice, with the objective of creating better working conditions, through quality management capable of guaranteeing efficient and safe care, aiming at an environment that attracts and retains professionals. To this end, it is reinforced the urgency of interdependent practices, actions that strengthen interprofessionality in care, implementation of a culture of systematic assessment and investments in professional training.

## CONCLUSIONS

In process, it was evident that theoretical and legal support of professional practice and care planning, evaluation and continuity presented statistically the highest scores, whereas interdependent practices in professional practice obtained the worst. Outcome was favorable to quality of care, with systematic assessment of nursing care and indicators being very favorable, but systematic assessment of nurses’ performance and supervision was moderately favorable. Different variables characterizing the nurses had an association and inverse association with process and outcome.

A large effect was obtained between process and a medium effect with outcome with the total score of job satisfaction. Hence, the set of findings brings advances in knowledge about the process and outcome dimensions present in work environments and the relationship with nursing professional satisfaction. Such contributions can help developing improvement strategies in these environments that have an impact on health promotion and disease prevention in these professionals, in addition to improving quality of care for patients and their families through the adaptation of work processes.
