# Supplementary material for: Nursing professional practice environments and job satisfaction: process and outcome interfaces
Source: Rev Bras Enferm. 2025 Jun 27;78(2):e20230544. doi: 10.1590/0034-7167-2023-0544 (PMC12208684; doi:10.1590/0034-7167-2023-0544)
Supplement: Translation

# Translation: Ambientes de prática profissional de enfermagem e satisfação no trabalho: interfaces do processo e resultado

**DOI:** 10.1590/0034-7167-2023-0544pt

**Authors:** Alessandro Rodrigues Perondi, Letícia de Lima Trindade, Olga Maria Pimenta Lopes Ribeiro, Jane Tavares Gomes, Jouhanna do Carmo Menegaz, Simone Coelho Amestoy, José Luis Guedes dos Santos, João Miguel Almeida Ventura-Silva

**Affiliations:** I Universidade Comunitária da Região de Chapecó. Chapecó, Santa Catarina, Brasil; II Escola Superior de Enfermagem do Porto. Porto, Portugal; III Universidade do Estado de Santa Catarina. Chapecó, Santa Catarina, Brasil; IV Universidade Federal do Vale do São Francisco. Petrolina, Pernambuco, Brasil; V Universidade Federal de Santa Catarina. Florianópolis, Santa Catarina, Brasil; VI Escola Superior de Saúde Norte da Cruz Vermelha Portuguesa. Oliveira de Azeméis, Portugal

**Keywords:** Enfermagem, Ambiente de Trabalho, Prática Profissional, Satisfação no Trabalho, Hospitais

## Abstract

**Objetivos:**

avaliar o processo e o resultado dos ambientes da prática profissional de enfermagem e sua relação com a satisfação no trabalho.

**Métodos:**

estudo transversal, realizado em oito hospitais no sul do Brasil. Participaram 291 enfermeiros que responderam ao questionário sociodemográfico/profissional, *Scale for the Environment Evaluation of Professional Nursing Practice* e Escala de Satisfação no Trabalho. Utilizou-se análise estatística descritiva e inferencial.

**Resultados:**

no processo, os subsídios teóricos/legais e planejamento, avaliação e continuidade do cuidado apresentaram os maiores escores, enquanto as práticas interdependentes obtiveram os piores. O resultado revelou-se favorável à qualidade dos cuidados, sendo a avaliação sistemática dos cuidados e dos indicadores muito favorável, mas a avaliação sistemática do desempenho e supervisão moderada. Obteve-se grande efeito do processo e médio do resultado no escore total da satisfação no trabalho.

**Conclusões:**

os elementos do processo e resultado indicam como promover a satisfação profissional e a qualidade da assistência de enfermagem.

## INTRODUÇÃO

A gestão da qualidade dos serviços de saúde tem sido tema de estudo ao longo dos anos, sendo considerado prioridade na busca pela melhoria da segurança do paciente e sustentabilidade organizacional. Este assunto torna-se um desafio para as lideranças de enfermagem, devido à complexidade envolvida nos ambientes da prática profissional e nos processos assistenciais^(1)^.

A expressão “ambiente da prática profissional de enfermagem” é utilizada internacionalmente para definir um conjunto de características organizacionais concretas e abstratas que facilitam ou restringem a prática profissional da enfermagem na entrega de um cuidado de alta qualidade^(2)^.

Reconhece-se que o enfermeiro e o ambiente de trabalho têm papel fundamental para garantia da segurança e qualidade dos cuidados prestados, reconhecendo que alguns atributos, tais como autonomia profissional, controle sobre a prática profissional do enfermeiro, relações profissionais cordiais entre médicos e enfermeiros e suporte organizacional, são elementos essenciais para a construção de um ambiente favorável ou desfavorável^(3)^.

A literatura evidencia que ambientes da prática profissional de enfermagem favoráveis têm sido associados a maior satisfação profissional^(4)^, aos baixos níveis de síndrome de *burnout* e exaustão emocional^(1)^, redução da intenção de abandonar o emprego^(5)^, melhora no clima de segurança e na qualidade dos cuidados de saúde^(6,7)^, redução da falta de cuidados de enfermagem^(8)^, menores taxas de absenteísmo e redução de iatrogenias^(3,6)^.

Como parte dos esforços para melhorar a segurança dos pacientes e a qualidade do cuidado, organizações profissionais como a *Institute of American* e a *Nurses Credentialing Cente*r (ANCC) têm enfatizado a promoção dos ambientes de trabalho da enfermagem^(3)^. A Organização Mundial da Saúde, no relatório sobre o estado da enfermagem no mundo, relembra a necessidade de os países proporcionarem ambientes favoráveis à prática de enfermagem, com o intuito de atrair, reter e motivar essa força de trabalho^(9)^, visto que esses profissionais são determinantes na qualidade e segurança dos cuidados prestados aos clientes, famílias e/ou cuidadores. Em maio de 2022, a *International Labor Organization* (ILO), no artigo 7º, incluiu as condições de trabalho seguras e saudáveis no seu quadro de princípios e direitos fundamentais no trabalho^(10)^.

De acordo com o modelo teórico de Donabedian^(11)^, alguns fatores contribuem para a melhoria da qualidade em saúde e, consequentemente, para a qualificação dos ambientes de prática, sendo que, para sua avaliação, estabelece-se uma tríade que consiste na estrutura, no processo e no resultado. A estrutura é entendida como recursos físicos, humanos, materiais, financeiros e equipamentos necessários para a assistência à saúde; o processo refere-se às atividades envolvendo profissionais de saúde e usuários, incluindo diagnóstico, tratamento, aspectos éticos de relação profissional; e o resultado corresponde ao produto final da assistência prestada, considerando a saúde, a satisfação de padrões e as expectativas dos usuários^(11)^. O processo se refere aos fatores relacionados ao desempenho das atividades inerentes à concepção e prestação de cuidados de enfermagem. O resultado avalia as mudanças desejáveis ou indesejáveis, relativamente aos cuidados, aos clientes e aos enfermeiros^(12)^.

Apesar do avanço na produção de conhecimento sobre esse tema, a literatura tem mostrado que a maioria dos estudos utiliza escalas de avaliação derivadas da *Nursing Work Index*, trazendo a limitação de estarem focados essencialmente nas condições estruturais da organização e superficialmente os componentes processo e resultado^(12)^.

Neste sentido, estudos do ambiente da prática de enfermagem que contemplem as dimensões processo e resultado surgem como alternativa para contribuir na compreensão dos aspectos que interferem no exercício da prática profissional e que, consequentemente, podem ter repercussões no cuidado ao paciente^(1)^, sendo também objeto de interesse a satisfação profissional. Esta corresponde à percepção dos profissionais sobre o trabalho que exercem e ao ambiente no qual ele é produzido^(13)^.

A satisfação no trabalho pode influenciar a qualidade da assistência prestada, reduzir o nível de *burnout*, diminuir a taxa de mortalidade e, consequentemente, diminuir a rotatividade e o absenteísmo, promover o interesse pelo trabalho e a qualidade das relações interpessoais^(14)^, com o impacto dos ambientes de trabalho saudáveis na satisfação e retenção dos enfermeiros focos de estudos na última década^(3,15,16)^.

No cenário brasileiro, entende-se pertinente desenvolver investigações que identifiquem diversos fatores, não apenas os organizacionais, que possam repercutir na prestação dos cuidados, com vistas a subsidiar a tomada de decisão das lideranças, na busca da melhoria contínua dos processos e melhores condições para o trabalho da equipe de enfermagem^(2)^.

## OBJETIVOS

Avaliar o processo e o resultado dos ambientes da prática profissional de enfermagem e sua relação com a satisfação no trabalho.

## MÉTODOS

### Aspectos éticos

O estudo foi aprovado pelo Comitê de Ética em Pesquisa Envolvendo Seres Humanos, e seguiu todas as orientações da Resolução nº 466/2012. Os participantes assinaram o Termo de Consentimento Livre e Esclarecido.

### Delineamento, período e local do estudo

Trata-se de estudo transversal e quantitativo, orientado pela ferramenta *STrengthening the Reporting of OBservational studies in Epidemiology* (STROBE). Foi realizado em oito hospitais da região Sul do Brasil, escolhidos intencionalmente, por se tratar de hospitais de referência na oferta de leitos públicos e no atendimento de média e alta complexidade.

### População e definição da amostra

Utilizou-se a amostragem não probabilística por conveniência, considerando uma heterogeneidade de 50%, Intervalo de Confiança de 95%, com erro amostral de 5%, que resultou em uma amostra mínima de 232 participantes, estratificada proporcionalmente por cada hospital. Contudo, a coleta de dados totalizou uma amostra de 291 enfermeiros (Quadro 1), considerando os critérios de inclusão: a) atuar como enfermeiro nos hospitais do estudo; b) possuir um período de experiência na unidade igual ou superior a três meses; c) estar atuando no período da coleta, ou seja, não estar afastado por qualquer motivo. Participaram do estudo enfermeiros de todos os setores das instituições selecionadas, que prestavam assistência direta aos pacientes, nos diferentes turnos de trabalho.

**Quadro 1:** Dados de identificação do cenário e quantitativo de participantes do estudo (N=232), Região Sul, Brasil, 2021

Identificação	População/enfermeiros	Amostra
Hospital A	68	27
Hospital B	28	11
Hospital C	39	16
Hospital D	40	16
Hospital E	179	71
Hospital F	77	30
Hospital G	129	51
Hospital H	25	10
**Total**	**581**	**232**

*Fonte: http://cnes.datasus.gov.br, março, 2021.*

### Instrumentos utilizados para coleta das informações

A coleta de dados ocorreu no período de julho a outubro de 2021, por meio de visitas *in loco* às instituições selecionadas. Foi utilizado questionário estruturado, composto por três partes. Na primeira, buscaram-se informações sobre características sociodemográficas e profissionais (idade, sexo, estado civil, formação profissional, tipo de instituição - pública ou privada, unidade de trabalho, condição profissional - assistência ou coordenação, tempo de exercício profissional, tempo de trabalho na instituição e na unidade).

A segunda parte continha o instrumento *Scale for the Environment Evaluation of Professional Nursing Practice* (*SEE-Nursing Practice*)^(12)^, adaptado linguisticamente para o português do Brasil para este estudo. O *SEE-Nursing Practice* é constituído por três subescalas: *SEE-Nursing Practice* - Estrutura; *SEE-Nursing Practice* - Processo; e *SEE-Nursing Practice* - Resultado.

Neste estudo, utilizaram-se a *SEE-Nursing Practice* - Processo e a *SEE-Nursing Practice* - Resultado. A *SEE-Nursing Practice* - Processo é composta por 37 itens, divididos em seis dimensões: 1 - colaboração e trabalho em equipe (9 itens); 2 - estratégias para a garantia da qualidade na prática profissional (7 itens); 3 - práticas autônomas no exercício profissional (7 itens); 4 - planejamento, avaliação e continuidade do cuidado (6 itens); 5 - suporte teórico e legal da prática profissional (4 itens) e 6 - práticas interdependentes na prática profissional (4 itens). A SEE-*Nursing Practice* - Resultado inclui 13 itens, divididos em duas dimensões: 1 - avaliação sistemática da assistência de enfermagem e indicadores (7 itens); 2- avaliação sistemática do desempenho e supervisão do enfermeiro (6 itens)^(12)^.

A consistência interna da versão brasileira obteve o coeficiente alpha de Cronbach de 0,95 para o *SEE-Nursing Practice* - Processo e 0,94 para o *SEE-Nursing Practice* - Resultado. A resposta é mensurada a cada item, em escala tipo Likert com cinco opções, em que um corresponde a “nunca”, dois, a “raramente”, três, a “às vezes”, quatro, a “muitas vezes”, e cinco, a “sempre”^(12)^.

A terceira parte do estudo utilizou a Escala de Satisfação no Trabalho (EST), versão reduzida^(15)^, que tem como objetivo identificar o grau de satisfação dos colaboradores em relação ao seu ambiente de trabalho. A EST apresenta 15 itens, e é subdividida em cinco dimensões, composta por três itens cada: satisfação com os colegas de trabalho (α = 0,86); satisfação com o salário (α = 0,92); satisfação com a chefia (α = 0,90); satisfação com a natureza do trabalho (α = 0,82) e satisfação com as promoções (α = 0,87). As respostas dos participantes são obtidas por meio de escala de sete pontos (1 = totalmente insatisfeito; 2 = muito insatisfeito; 3 = insatisfeito; 4 = indiferente; 5 = satisfeito; 6 = muito satisfeito; 7 = totalmente satisfeito), sendo que a análise dos resultados da escala se dá através do escore/pontuação médio de cada uma das cinco dimensões^(17)^.

### Tratamento e análise dos dados

Os dados coletados foram analisados através do programa *Statistical Package for the Social Sciences* (SPSS), versão 21.0. As variáveis categóricas foram descritas por frequências absolutas e relativas. Em relação à *SEE-Nursing Practice* e suas subescalas, quanto mais elevada for a pontuação, mais o processo e o resultado são favoráveis à qualidade dos cuidados. Para análise dos resultados, foram descritos a média e o desvio padrão dos itens, e utilizaram-se os critérios a seguir^(12)^: pontuação < 35% - componente do ambiente da prática profissional de enfermagem pouco favorável à qualidade dos cuidados; entre 35% e 55% - componente do ambiente da prática profissional de enfermagem moderadamente favorável à qualidade dos cuidados; entre 55% e 75% - componente do ambiente da prática profissional de enfermagem favorável à qualidade dos cuidados; e > 75% - componente do ambiente da prática profissional de enfermagem muito favorável à qualidade dos cuidados.

Para a expressão do resultado da EST, considerou-se que, quanto maior o valor do escore médio, maior o grau de contentamento ou satisfação do colaborador com aquela dimensão. Os valores entre 5 e 7 indicam satisfação; valores entre 1 e 3,9 indicam insatisfação; e valores entre 4 e 4,9 indicam indiferença^(17)^.

Para comparar médias, os testes t de *Student* para amostras independentes ou Análise de Variância (ANOVA) foram aplicados. As associações entre as variáveis contínuas foram avaliadas pelos testes da correlação de Pearson ou Spearman, considerando valores do coeficiente de correlação abaixo de 0,3 como de pequeno efeito, entre 0,30 e 0,49, médio efeito, e entre 0,50 e 1, grande efeito. Para controle de fatores confundidores, a análise de Regressão Linear Multivariada foi utilizada. Foram calculados o coeficiente de regressão ou angular (b), que mede o efeito no desfecho a cada aumento de uma unidade do fator, e o Intervalo de Confiança de 95%. O critério para a entrada da variável no modelo foi de que a mesma apresentasse um valor de p<0,20 na análise bivariada. O nível de significância adotado foi de 5% (p≤0,05).

## RESULTADOS

Participaram do estudo 291 enfermeiros, dos quais 251 (86,3%) eram do sexo feminino e 216 residiam com companheiro (74,2%). O nível de escolaridade demonstrou que 176 (60,5%) possuíam especialização, 100 (34,4%) eram apenas graduados e 15 (5,1%) apresentavam mestrado ou doutorado. A média de idade foi de 34,2 ± 8,4 anos; a mediana do tempo de exercício na profissão foi de seis anos (2 - 12); e a mediana do tempo de exercício no atual serviço foi de dois (1 - 7).

Houve maiores prevalências de enfermeiros que exerciam suas atividades em instituições públicas (n=114/39,2%), precedidas por instituições filantrópicas (n=101/4,7%) e privadas (n=76/26,1%). Desses, 212 (72,9%) atuavam na assistência e 79 (27,1%) tinham sob sua responsabilidade, além da assistência, o cargo de coordenação. Na Tabela 1, é apresentado o resultado da avaliação da dimensão processo nos ambientes pesquisados.

**Tabela 1:** Avaliação da dimensão processo dos ambientes da prática profissionais de enfermagem, Região Sul, Brasil, 2021

Processo	Média ± DP	PF^*^	MDF^*^	F^*^	MTF^*^
		n (%)	n (%)	n (%)	n (%)
Colaboração e trabalho em equipe	68,6 ± 16,8	8 (2,7)	48 (16,5)	145 (49,8)	90 (30,9)
Estratégias para garantia da qualidade dos cuidados	65,1 ± 18,1	15 (5,2)	71 (24,4)	130 (44,7)	75 (25,8)
Práticas autônomas no exercício profissional	68,2 ± 15,2	4 (1,4)	53 (18,2)	160 (55,0)	74 (25,4)
Planejamento, avaliação e continuidade do cuidado	71,4 ± 16,1	6 (2,1)	44 (15,1)	135 (46,4)	106 (36,4)
Subsídios teóricos e legais para o exercício da profissional	78,9 ± 15,1	2 (0,7)	18 (6,2)	133 (45,7)	138 (47,4)
Práticas interdependentes no exercício profissional	31,1 ± 17,5	171 (58,8)	94 (32,3)	24 (8,2)	2 (0,7)
Total	65,4 ± 11,6	2 (0,7)	57 (19,6)	170 (58,4)	62 (21,3)

* *PF - pouco favorável; MDF - moderadamente favorável; F - favorável; MTF - muito favorável.*

A tabela revela que os subsídios teóricos e legais para o exercício da profissional (média=78,9 ± 15,1) e o planejamento, avaliação e continuidade do cuidado (média=71,4 ± 16,1) foram os itens que apresentaram estatisticamente os maiores escores. O pior escore foi observado nas práticas interdependentes no exercício profissional (31,1 ± 17,5). A avaliação geral do processo apresentou-se favorável à qualidade dos cuidados (média=65,4 ± 11,6).

A Tabela 2 apresenta os escores médios da dimensão resultado presente nos ambientes da prática profissionais de enfermagem pesquisados.

**Tabela 2:** Avaliação da dimensão resultado dos ambientes da prática profissionais de enfermagem, Região Sul, Brasil, 2021

Resultado	Média ± DP	PF^*^	MDF^*^	F^*^	MTF^*^
		n (%)	n (%)	n (%)	n (%)
Avaliação sistemática dos cuidados e dos indicadores de enfermagem	64,2 ± 19,8	22(7,6)	75 (25,8)	125(43,0)	69 (23,7)
Avaliação sistemática do desempenho e supervisão dos enfermagem	53,2 ± 21,7	65 (22,3)	99 (34,0)	88 (30,2)	39 (13,4)
Total	59,1 ± 19,3	35 (12,0)	86 (29,6)	116(39,9)	54 (18,6)

* *PF - pouco favorável; MDF - moderadamente favorável; F - favorável; MTF - muito favorável.*

A avaliação sistemática dos cuidados e dos indicadores de enfermagem apresentou maior escore (média=64,2 ± 19,8). Já a avaliação sistemática do desempenho e supervisão dos enfermagem (média=53,2 ± 21,7) se mostrou moderadamente favorável, contudo a avaliação geral da dimensão resultado apresentou-se também favorável à qualidade dos cuidados (média=59,1 ± 19,3).

Identificou-se associação entre as variáveis de caracterização da amostra e os escores das dimensões processo e resultado nas variáveis sexo (p=0,267 e p=0,920), estado civil (p=0,991 e p=0,720), grau acadêmico (p=0,190 e p=0,024), tipo de instituição (p=0,979 e p=0,824) e condição profissional (p=0,306 e p=0,761).

Associações inversas e estatisticamente significativas do processo e resultado foram observadas com as variáveis idade (-0,171; p=0,003 e -0,174; p=0,003), tempo de exercício na profissão (-0,217; p<0,001 e -0,217; p<0,001) e também no tempo de exercício no serviço (-0,185; p<0,002 e -0,153; p<0,009), respectivamente. Demonstrou-se que, quanto maior a idade e o tempo na profissão e no serviço, menor o escore das dimensões processo e resultado. Não obstante, também foi revelada uma associação significativa entre o grau acadêmico e a dimensão resultado, sendo que, à medida que aumenta o nível de escolaridade, diminui o escore dessa dimensão.

Para controle de fatores de confusão, as variáveis com p <0,20 foram inseridas em um modelo de Regressão Linear Multivariado. Após o ajuste pelo modelo multivariado, o tempo de exercício na profissão permaneceu um fator independente associado ao processo e ao resultado, sendo que, quanto maior o tempo de exercício na profissão, menores são os escores nessas dimensões (Tabela 3).

**Tabela 3:** Caracterização das variáveis segundo o modelo de Regressão Linear Multivariado, Região Sul, Brasil, 2021

Variáveis	Processo		Resultado	
	b (IC 95%)	Valor de *p*	b (IC 95%)	Valor de *p*
Idade (anos)^*^	0,03 (-0,20 a 0,25)	0,822	0,02 (-0,35 a 0,40)	0,900
Grau acadêmico				
Graduação	0,00	-	0,00	-
Especialização	-0,13 (-3,13 a 2,87)	0,932	-1,94 (-6,90 a 3,02)	0,442
Mestrado/doutorado	-0,80 (-7,29 a 5,70)	0,809	-6,59 (-17,3 a 4,17)	0,229
Tempo de exercício na profissão (anos)^**^	-0,38 (-0,72 a -0,05)	0,025	-0,65 (-1,21 a -0,10)	0,022
Tempo de exercício no serviço (anos)^**^	-0,06 (-0,41 a 0,29)	0,720	0,06 (-0,52 a 0,64)	0,836

* Coeficiente de correlação de Pearson;

** Coeficiente de correlação de Spearman.

Em relação à EST, o escore geral demonstrou indiferença (média 4,60 ± 0,78). Os escores das dimensões isoladas demonstraram satisfação com a chefia (média 5,34 ± 1,06), com os colegas (média 4,94 ± 0,87) e com a natureza do trabalho (média 4,91 ± 0,84), indiferença com as promoções (média 4,20 ± 1,19) e insatisfação com o salário (média 3,58 ± 1,32).

A Tabela 4 apresenta a associação entre os itens com p≤0,001 da EST com as dimensões processo e resultado da *SEE-Nursing Practice*. Pôde-se observar grande efeito na dimensão processo e médio efeito na dimensão resultado, com o escore total da EST.

**Tabela 4:** Correlação entre a Escala de Satisfação no Trabalho e as dimensões processo e resultado da *SEE-Nursing Practice* através do coeficiente de correlação de Pearson, Região Sul, Brasil, 2021

Dimensões da Escala de Satisfação no Trabalho	*SEE-Nursing Practice*	
	Processo	Resultado
Satisfação com os colegas	0,304^b^	0,192^a^
Satisfação com o salário	0,310^b^	0,404^b^
Satisfação com a chefia	0,341^b^	0,270^a^
Satisfação com a natureza do trabalho	0,364^b^	0,284^a^
Satisfação com as promoções	0,449^b^	0,435^b^
Total	0,553^c^	0,490^b^

a pequeno efeito;

b médio efeito;

c grande efeito.

## DISCUSSÃO

O interesse dos pesquisadores em explorar e melhorar o ambiente da prática profissional de enfermagem não é novo. A *American Association of Critical Care Nurses* publicou e revisou as normas para estabelecer e manter ambientes de trabalho saudáveis, atualizadas em 2016^(16)^. Desde então, a literatura^(18,19)^ tem demonstrado que um ambiente da prática profissional de enfermagem favorável constitui um fator determinante para a qualidade da assistência, bem como para a obtenção de melhores resultados para os clientes, para os enfermeiros e para as instituições, o que culmina na sua priorização neste último ano pela ILO^(9)^.

Os achados demográficos e laborais revelaram um perfil semelhante a outras pesquisas no contexto nacional e internacional^(12)^ e a estudo realizado no país pelo Conselho Federal de Enfermagem^(20)^.

O estudo mostrou que 60,5% dos enfermeiros possuíam especialização, e este achado apresentou associação significativa com a dimensão resultado, sendo que, à medida que aumenta o nível de escolaridade, diminui o escore dessa dimensão. Essa dimensão avalia as mudanças desejáveis ou indesejáveis, relativamente aos cuidados, aos clientes, bem como aos enfermeiros^(11)^. A elevação no grau de escolaridade tem sido associada à diminuição da taxa de mortalidade em hospitais da Bélgica^(21)^.

Níveis elevados de escolaridade relacionam-se positivamente à produtividade, uma vez que a educação contribui na elevação do conhecimento, habilidades, atitudes e comportamentos essenciais para a realização das atividades da prática de enfermagem^(22)^. A educação superior está relacionada à capacidade de melhor avaliar uma situação antes de agir^(23)^, sendo o investimento na formação um aspecto claramente importante para promover a qualidade dos resultados na assistência de enfermagem, o que também tem potencial de elevar a criticidade e exigências dos trabalhado. Estudo discute ainda a dificuldade para que a formação garanta uma prática continuamente alicerçada em evidências; esta passa pela capacidade de pesquisar e abertura para utilizar as evidências na prática, o que também contribui para mudança nos ambientes^(24)^.

Os profissionais possuíam uma mediana de seis anos de tempo de exercício na profissão e de dois anos de experiência no atual serviço, características semelhantes a outros estudos^(2,14)^, corroborando ainda os dados impressos pelo perfil da enfermagem no Brasil^(20)^.

O tempo de exercício na profissão apresentou associação inversa e estatisticamente significativas com a dimensão processo e resultado, sendo que, quanto maior o tempo de exercício na profissão, menores são os escores nessas dimensões. O desgaste, ao longo dos anos, e a complexidade do exercício profissional, durante a pandemia, podem ter contribuído para que os participantes ficassem com pior percepção das atividades realizadas e pior produto final da assistência prestada. Em contraponto, a experiência de trabalho é um processo de desenvolvimento de perícia, na medida em que, quanto mais vivências no trabalho, mais oportunidades se têm de adquirir habilidades técnicas e outras para desempenhar suas tarefas de forma eficaz. A experiência laboral também contribui para um ambiente de trabalho positivo, incluindo o aumento da segurança do paciente^(25)^. Nessa direção, emergem a educação continuada e a educação permanente nos serviços que precisam considerar que, nos contextos assistenciais, atuam diferentes gerações de trabalhadores e o próprio envelhecimento da força de trabalho. Um ambiente que capacite e motive os enfermeiros é necessário para sustentar a força de trabalho de enfermagem, com fomento da autonomia nas decisões e preocupação com o esgotamento da força de trabalho^(15)^. Saber somar os saberes dos enfermeiros experiências e as inovações trazidas pelos ingressantes na profissão constitui-se desafio da profissão no contexto atual.

As instituições públicas e/ou filantrópicas abarcavam 73,9% dos profissionais envolvidos no estudo. Segundo pesquisadores^(25)^ no Brasil, o setor público tem despontado como o maior e mais importante empregador da categoria, visto que 58,9% da força de trabalho da enfermagem está vinculada a esse setor. Contudo, cabe destacar que as características do ambiente dos hospitais públicos e/ou financiados pelo Sistema Único de Saúde brasileiro se mostraram precárias, quando comparadas às dos hospitais privados^(26)^.

O subfinanciamento dos hospitais públicos pode contribuir para que características como serviços de apoio, programas de educação permanente, dimensionamento de pessoal e desenvolvimento na carreira não estejam adequadas nessas instituições^(25)^.

Os participantes atuavam predominantemente na assistência (72,9%), semelhante aos achados de outro estudo em três hospitais universitários das regiões Sul e Sudeste do Brasil^(27)^ e de investigação em hospital universitário de São Paulo^(24)^. A enfermagem é uma ciência e profissão essencial, considerada nuclear na estrutura das profissões de saúde no Brasil e no mundo. Essa noção sociológica de essencialidade possibilita atuar nas várias dimensões da saúde, com valorização muito forte da assistência^(25)^.

A desvalorização do papel da enfermagem tem sido constantemente apontada por órgãos internacionais. Os profissionais de enfermagem são frequentemente mal renumerados e vítimas de desigualdade de gênero e de precárias condições de trabalho^(28)^, com implicações na satisfação profissional e na tendência de rotatividade e abandono da profissão^(15)^.

A avaliação geral da dimensão processo demonstrou-se favorável à qualidade dos cuidados. Nesta dimensão, são avaliados os fatores relacionados ao desempenho das atividades inerentes à concepção e prestação de cuidados de enfermagem^(12)^. O item relacionado às práticas interdependentes no exercício profissional apresentou o pior escore. A interdependência é observada na necessidade de um conjunto de trabalhadores atuar em conjunto para atingir um objetivo comum. Considera-se que um ambiente desfavorável às práticas interdependentes indicia para as fortes relações de poder ainda marcantes nos hospitais e para a existência de modelos assistenciais que pouco favorecem a interprofissionalidade.

Relações conflituosas entre os profissionais, especialmente pela centralidade na valorização dos saberes médicos, têm sido descritas em diversos estudos^(2,13)^, com repercussões na insatisfação no trabalho e, consequentemente, negativas para a qualidade do ambiente de trabalho.

Por outro lado, o escore geral do processo demonstrou associação significativa ao escore total da EST, apontando que ambientes que apresentam melhores avaliações sobre a dimensão processo apresentam melhor satisfação geral com o trabalho. A autonomia profissional e relações harmoniosas são as práticas com maiores preditores positivos no favorecimento da prática profissional do enfermeiro e na melhora da satisfação em seu ambiente de trabalho^(15,29)^.

Na análise da dimensão resultado, a avaliação geral se apresentou também como favorável à qualidade dos cuidados. Essa dimensão avalia as mudanças desejáveis ou indesejáveis, relativamente aos cuidados, aos clientes, bem como aos enfermeiros^(12)^. Esse fator é composto por dois itens, como a avaliação sistemática dos cuidados e dos indicadores de enfermagem e a avaliação sistemática do desempenho e supervisão dos enfermeiros.

A avaliação sistemática deve ser entendida como um instrumento gerencial do enfermeiro, que tem o potencial de gerar transformações por meio de processos graduais e contínuos de mudanças, visando à integralidade da assistência e à ampliação da participação profissional^(30)^. Contudo, dificuldades no cumprimento de atribuições privativas, como a gestão do cuidado e processo de enfermagem, são relacionadas ao dimensionamento de pessoal de enfermagem, com implicações no absenteísmo, sendo aspectos fundamentais para a qualidade do ambiente profissional da enfermagem^(28,31)^.

Nessa direção, também se reforça a importância do dimensionamento de pessoal de enfermagem, com aspectos importantes no que se refere ao quantitativo e também aos aspectos qualitativos da força de trabalho, uma vez que a qualidade e a continuidade dos cuidados dependem da disponibilidade da força de trabalho e também da sua capacidade técnica para o trabalho, ou seja, da sua formação profissional, que envolve investimento na educação continuada e permanente nas instituições.

As características e os atributos identificados na prática de enfermagem, incluindo a ênfase na aprendizagem contínua e nas relações interpessoais, têm impacto profundo na enfermagem. Os enfermeiros precisam se mostrar adaptáveis, compassivos e centrados no paciente em um ambiente de saúde em constante evolução^(32)^.

Nessa direção, também cabe mencionar a crescente exigência do domínio das tecnologias em saúde em franca expansão, que aumentam as exigências no trabalho dos enfermeiros, exigem remover enfermeiros das áreas diretas de atendimento ao paciente e fazem com que alguns enfermeiros experientes lutem com a tecnologia e abandonem a profissão pela dificuldade de domínio das tecnologias^(15)^. Essas podem ser geradoras de satisfação e insatisfação profissional, bem como mudam a interação profissional e paciente.

A satisfação geral demonstrou indiferença, contudo a análise das dimensões isoladas demonstrou insatisfação com o salário. Pesquisadores têm discutido a remuneração como domínio mais importante para a satisfação profissional da equipe de enfermagem^(13)^. A instituição de um piso salarial para a enfermagem encontra-se em tramitação por meio do Projeto de Lei nº 2.564 de 2020 e do Projeto de Emenda à Constituição nº 11 de 2022, ambos recentemente aprovados pelo Senado e pela Câmara Federal, mas com entraves para sua implantação. A ausência de um piso salarial e de uma jornada de trabalho semanal definida por lei induz o processo de trabalho da equipe ser organizado por ritmos acelerados, extensas jornadas laborais, acúmulo de funções, variados vínculos empregatícios, fragmentação das práticas assistenciais, desvalorização social, baixos salários e submissão às condutas repressoras e autoritárias de uma hierarquia rígida e vertical^(33)^.

Os achados indicaram também satisfação com a chefia, com os colegas de trabalho e com a natureza do trabalho. Uma boa relação com colegas de trabalho e a chefia é conhecidamente determinante para a satisfação no trabalho. A satisfação com a chefia está relacionada ao grau de reconhecimento da capacidade profissional do chefe pelo seu subordinado. Já a satisfação com os colegas de trabalho reflete o quanto o trabalhador está de acordo com a colaboração, a amizade, a confiança e o relacionamento estabelecido com os demais^(17)^.

É válido destacar que as atividades/tarefas do enfermeiro hospitalar são caracterizadas em assistências administrativas e gerenciais, sobretudo no que tange à execução de procedimentos, pois proveem recursos materiais e humanos, planejam e supervisionam a assistência executada pelos técnicos de enfermagem, descrevem rotinas, organizam o processo de trabalho e promovem a educação em serviço^(34,35)^. Contudo, as prioridades organizacionais sofrem mudanças durante o período pandêmico^(36)^. A satisfação com esse item pode sinalizar que os ambientes de estudo oferecem as condições necessárias para o desempenho das atividades com qualidade e segurança.

### Limitações do estudo

O estudo possui limitações, como a coleta de dados, desenvolvida em um contexto de pandemia, o que pode ter influenciado a resposta dos participantes, sendo relevantes investigações longitudinais que avaliem sistematicamente os ambientes de prática profissional de enfermagem, além da satisfação profissional dos enfermeiros.

### Contribuições para a área da enfermagem

Os achados indiciam as dimensões que comprometem a qualidade dos ambientes de prática de enfermagem nos hospitais. Reforçam a necessidade de ações que favoreçam a prática profissional do enfermeiro, com o objetivo de criar melhores condições de trabalho, por meio de uma gestão de qualidade capaz de garantir um atendimento eficiente e seguro, objetivando um ambiente que atraia e retenha seus profissionais. Para isso, reforça-se a urgência das práticas interdependentes, de ações que fortaleçam a interprofissionalidade na assistência, a implantação da cultura de avaliação sistemática e de investimentos na formação profissional.

## CONCLUSÕES

No processo, evidenciou-se que os subsídios teóricos e legais para o exercício da profissional e o planejamento, avaliação e continuidade do cuidado apresentaram estatisticamente os maiores escores, enquanto que as práticas interdependentes no exercício profissional obtiveram os piores. O resultado revelou-se favorável à qualidade dos cuidados, sendo a avaliação sistemática dos cuidados e dos indicadores muito favorável, mas a avaliação sistemática do desempenho e supervisão foi moderadamente favorável. Diferentes variáveis de caracterização dos enfermeiros tiveram associação e associação inversa ao processo e resultado.

Obteve-se grande efeito entre o processo e médio efeito com o resultado com o escore total da satisfação no trabalho. Nesse sentido, o conjunto dos achados traz avanços no conhecimento sobre as dimensões processo e resultado presentes no ambiente de trabalho e a relação com a satisfação do profissional enfermeiro. Tais contribuições podem auxiliar na elaboração de estratégias de melhoria nesses ambientes que repercutam na promoção da saúde e prevenção do adoecimento desses profissionais, além de aprimorar a qualidade da assistência ao paciente e seus familiares por meio da adequação dos processos de trabalho.
